# The moderating role of gender on the relationship between childhood attention deficit and hyperactivity symptoms and functional impairment

**DOI:** 10.1371/journal.pone.0321767

**Published:** 2025-04-16

**Authors:** Zehra Koyuncu, Kübra Aslantürk Halil, Özge Selçukoğlu Kilimci, Büşra Arslan, Ezgi Tanrıöver Aydın, Emine Yücel, Yekta Uğur Arvas, Nazan Ebru Aksu, Cana Aksoy Poyraz, Ömer Faruk Demirel, Leyla Küçük, Burak Doğangün, Margaret Danielle Weiss, Mahmut Cem Tarakçıoğlu

**Affiliations:** 1 Department of Child and Adolescent Psychiatry, Cerrahpasa Faculty of Medicine, Istanbul University-Cerrahpasa, Istanbul, Turkey; 2 Florence Nightingale Faculty of Nursing, Istanbul University-Cerrahpasa, Istanbul, Turkey; 3 Department of Psychiatry, Cerrahpasa Faculty of Medicine, Istanbul University-Cerrahpasa, Istanbul, Turkey; 4 Department of Psychology, Selçuk University, Konya, Turkey; 5 Cerrahpasa Faculty of Medicine, Istanbul University-Cerrahpasa, Istanbul, Turkey; 6 Cambridge Health Alliance, Harvard Medical School, Cambridge, Massachusetts, United States of America; Chiba Daigaku, JAPAN

## Abstract

This study aimed to examine the link between childhood attention deficit and hyperactivity symptoms (CAS) and functional impairment in university students, while also investigating whether gender moderates this relationship. Six hundred and eighty university students participated in this cross-sectional study. The assessment was conducted using the Wender–Utah Rating Scale-25 (WURS), the Brief Symptom Inventory (BSI), and the Weiss Functional Impairment Rating Scale – Self Report (WFIRS-S). The relationship between CAS and general and domain-based functional impairment was evaluated using eight moderation models. Control variables, including age and concomitant psychiatric symptoms (five BSI scores), were added to the models. We observed positive associations between WURS and all WFIRS-S scores. In addition, WURS significantly interacted with gender in explaining WFIRS-S total (t = –2.26, p =.024). Gender also moderated the link between WURS and impairments in social (t = –2.00, p =.046) and risk domains (t = –2.86, p =.004). Accordingly, the associations between CAS and overall functional impairment, as well as impairments in social and risk domains, were stronger in men than in women. These findings highlight the significant role of CAS in functional impairments among university students, with gender emerging as a key moderating factor, particularly in social and risk-related domains.

## Introduction

Attention deficit/hyperactivity disorder (ADHD) is a common neurodevelopmental disorder. Up to 6% of people under the age 18 have a diagnosis of ADHD, and up to 3% of adults have or have had a diagnosis of ADHD [1]. ADHD symptoms persist into adulthood in 65% of individuals diagnosed in childhood [2] and continue to cause functional impairments [3]. Due to ADHD symptoms, both child and adult patients may experience problems in school, work, interpersonal relations including family, as well as in social activities [4,5]. While adult ADHD is common and impairing, it can be treated effectively [6]. However, undiagnosed or untreated patients exhibit considerable impairment in multiple domains [3,4,7].

ADHD symptoms and their impact on functioning vary depending on gender. ADHD is more common in men than in women [1], although some studies have indicated that the diagnosis of ADHD can be missed in women due to fewer externalizing symptoms [8–10]. Men and women may also experience significant functional impairment in different areas due to ADHD. For instance, while executive function deficits are similar between men and women [11], behavioral, educational, and occupational difficulties are more prevalent among men [9,11]. However, women may experience more difficulties in certain areas, such as social functioning, coping with stress, and time management [9,12]. In summary, evidence suggests that gender influences the outcomes of ADHD symptoms. Therefore, gender-related differences should be considered in the evaluation and management of ADHD [9].

There are conflicting findings in the literature regarding the variability of some features of ADHD across cultures. While significant cross-cultural variability was found in a study conducted in 42 countries [13], another reported similar diagnostic criteria and ADHD outcomes among adult ADHD patients. Notably, the latter study had some methodological limitations, including a small sample size and sample selection bias [14]. In addition, different gender-related functional outcomes of ADHD have been reported in studies conducted in different countries [9,11,12]. However, to the best of our knowledge, differences in functional impairment and ADHD outcomes between genders have not been investigated in a Turkish population.

University students were targeted in this study. University students who show symptoms of or have been diagnosed with ADHD constitute a unique group in terms of the challenges they face at their age and the cognitive demands of attending college. It has been suggested that young adults with ADHD who are enrolled in universities tend to function better than the general ADHD population [15].

Mental health problems, including anxiety disorders, depression, and ADHD symptoms, are commonly reported during the college years [16]. Furthermore, mental health problems have been found to be associated with functioning in various areas [17,18]. Therefore, we screened for current psychiatric symptoms, including them as control variables. Specifically, this study aimed to investigate the relationships between ADHD symptoms and functional impairments in a sample of university students from Turkey, while also assessing the moderating effect of gender on these relationships, with current psychiatric symptoms controlled for. Therefore, with the aim of contributing to the understanding of gender-related differences, this study explored the role of gender in functional impairments associated with ADHD in a sample from Turkey.

The following hypotheses guided this study

H1. There is a positive relationship between ADHD symptoms and functional impairment.

H2. Gender moderates the relationship between ADHD symptoms and functional impairment.

## Materials and methods

### Study design and variables

A two-center cross-sectional survey-based study was conducted to investigate the moderating role of gender on the relationship between ADHD symptoms and functional impairment among university students. The independent variable was ADHD symptoms, while the dependent variables included total functional impairment and its different subdomains (i.e., family, work, school, life skills, self-concept, social, and risk). The Wender–Utah Rating Scale-25 (WURS) was utilized as a screening tool to assess ADHD symptoms across a broad developmental period and symptom range [19]. Throughout the article, ADHD symptoms are referred to as childhood attention deficit and hyperactivity symptoms (CAS). Somatization, depression, anxiety, hostility, and self-concept were screened and included as control variables, making it possible to control for the effects of these psychiatric symptoms in students without an autism spectrum disorder (ASD), bipolar disorder (BD), or any psychotic disorder (PD) diagnosis.

### Setting

This research study was carried out during the 2021–2022 academic year and involved 1087 undergraduate students from the Istanbul University-Cerrahpasa, Florence Nightingale Faculty of Nursing and 2051 students from the Istanbul University-Cerrahpasa, Cerrahpasa Faculty of Medicine. Both nursing and medical students are considered undergraduate students in Turkey. The study was conducted in the students’ classrooms after obtaining the necessary permissions from the faculty boards. The purpose and methodology of the study were clearly explained to the students, and they were invited to participate. Email addresses were collected from participants for communication and for referral to the psychiatry outpatient clinic if their CAS scores exceeded the clinical threshold on the WURS. The scales were administered by four of the researchers, who were present in the classroom to provide any necessary assistance. The data were collected with an online survey tool. All surveys were consolidated into a single link for access, which was shared with students through tools such as the smartboard in the classroom. The link was sent via email or text message to those who preferred this method, allowing them to access it through their devices (e.g., tablets or mobile phones).

### Participants

Participation in the study was voluntary basis, and participants did not receive any compensation for their participation. Exclusion criteria included an antipsychotic treatment history and a diagnosis of ASD, BD, or PD due to the possibility of disruption in reality testing. Students with subclinical symptoms were not excluded, as their symptoms were screened and controlled. Additionally, students currently undergoing treatment for ADHD were excluded because such treatment can be related to functional outcomes.

**Fig 1:**
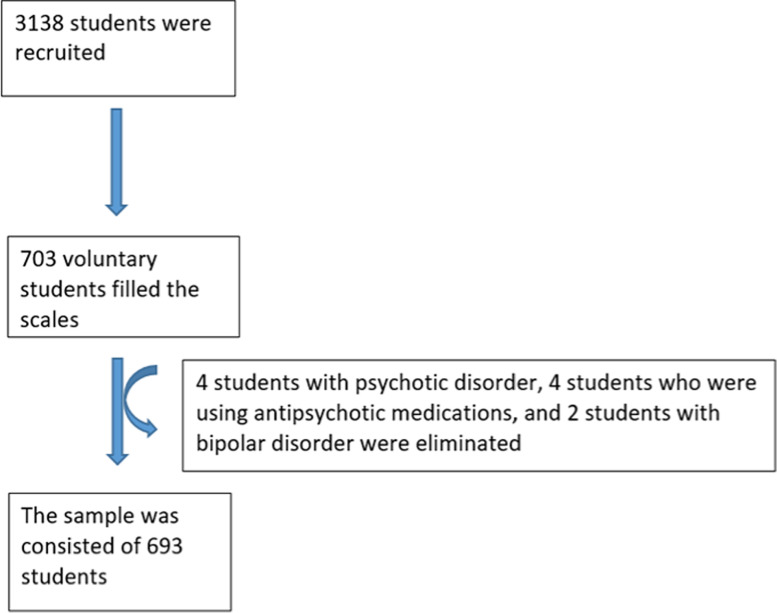
Flowchart of participant selection.

A total of 703 students participated in the study. Four were excluded because they met criteria for psychosis, and two were excluded because of a diagnosis of BD. An additional four students were excluded because they were taking antipsychotic medications. Therefore, the study group consisted of 693 students. (Fig 1) The flowchart illustrates the process of selecting the student sample, inclusion and exclusion criteria, and the characteristics of the final study group.

### Measurement tools

In this sample, ADHD symptoms were assessed using WURS [17], and functional impairment was assessed through the Weiss Functional Impairment Rating Scale: Self-report (WFIRS-S) [20]. Psychiatric symptoms were screened using the Brief Symptom Inventory (BSI) [21].

#### Sociodemographic data form.

A data form was created by the researchers to collect sociodemographic information, including the participant’s, age, gender, education, status of medical treatment/treatment history, and presence of ADHD diagnosis in childhood. There were two options for reporting gender: man and woman.

#### Brief symptom inventory (BSI).

BSI is a self-report instrument that is a shortened version of the Symptoms Checklist-90 [22]. The instrument was standardized previously with a sample of Turkish university students and shown to be a reliable instrument, with Cronbach’s alpha coefficient ranging from 0.63 to 0.86. The inventory consists of 53 items covering five symptom dimensions: somatization, depression, anxiety, hostility, and self-concept [21]. Respondents rate each feeling item on a five-point scale ranging from 0 (not at all) to 4 (extremely). A higher score indicates greater intensity of distress during the past seven days.

#### Weiss functional impairment rating scale – self report (WFIRS-S).

WFIRS-S is a self-report scale consisting of 69 items that assesses impaired functioning over the past month using a four-point Likert rating scale ranging from 0 (never or not at all) to 3 (very often, very much) [23]. WFIRS-S collects the perspectives of patients across seven domains: The family subdomain has eight items assessing issues related to family interactions, such as “Having problems with family”; The work subdomain has 11 items evaluating challenges in work or job settings, with items like “Problems with getting your work done efficiently”; The school subdomain has 10 items measuring academic performance and related difficulties, for example, “Problems meeting minimum requirements to stay in school”; The life skills subdomain has 12 items assessing the ability to manage daily living tasks, such as “Problems keeping up with household chores”; The self-concept subdomain has five items evaluating self-perception and confidence, with items like “Feeling incompetent”; The social activities subdomain includes nine items focused on engagement in social interactions and hobbies, such as “Problems participating in hobbies”; Finally, the risky activities subdomain has 14 items assessing engagement in impulsive or dangerous behaviors, for instance, “Breaking or damaging things.” The validity and reliability of the Turkish version of WFIRS-S was evaluated by Yalin-Sapmaz et al. The Cronbach’s alpha coefficient of the total scale was 0.94, and the test–retest reliability was acceptable.

In this study, statistical analyses were conducted using WFIRS-S subscale mean scores and WFIRS-S total mean scores. Mean scores were calculated by summing individuals scores and dividing by the number of items. Items marked “not applicable” were not included in the computation of the overall score. A higher score indicates greater impairment [20].

#### Wender-utah rating scale-25 (WURS).

WURS was developed in 1993 by Ward et al. [24] and adapted to Turkish by Oncu et al. [25]. It assesses ADHD symptoms using a Likert-type five-point scale ranging from “not at all/very slightly” (0) to “very much” (4), giving a possible range of 0–100 points. A higher score indicates higher ADHD symptoms [25]. WURS was chosen in this study for assessing ADHD symptoms because of its excellent discriminatory features and ability to screen for a wide variety of symptoms [19]. For the Turkish version, the Cronbach’s alpha coefficient for the total scale was 0.93 [25].

### Ethical standards

The procedures used in this study adhered to the tenets of the Declaration of Helsinki. The Istanbul University-Cerrahpasa, Cerrahpasa Medical School Ethics Committee approved all study materials (06.04.2021, E-67665420-604.02.01-69040). Informed consent was obtained from the participants who agreed to participate in the study.

### Statistical analysis

The initial dataset was screened for variable ranges, response patterns, outliers, and missing values. Four participants were excluded for careless or constant response patterns, six participants as univariate outliers, and three participants as multivariate outliers. Ultimately, 680 individuals were included in the analysis set. The scores for seven participants on the WFIRS-S work dimension and three on the WFIRS-S risk dimension could not be computed because participants selected the N/A option for all questions. As they constituted less than 5% of the cases, the mean substitution technique was used [26], wherein the means of the series were calculated and used to fill in the missing values for the relevant variables.

To evaluate our hypotheses, we performed a moderation analysis, which relies on normality in the distribution of variables due to its regression-based framework. To assess normality, we examined the skewness and kurtosis of the study variables. The values fell within the acceptable range of +/–2, suggesting univariate normality [27], except for the WFIRS-S risk scores. Nevertheless, we did not apply any transformation to this variable and used it in its original form, as deviations from normality tend to have a minimal impact on results in large samples [26]. We checked the dataset for multicollinearity by examining VIF and tolerance values, as strong correlations between predictor variables can weaken their individual contribution to predicting the dependent variable [26]. The results showed that all VIF values were under 5 [28], and tolerance values exceeded .10 [26], suggesting that multicollinearity was not a concern.

Prior to the main analysis, we computed descriptive statistics (including means, standard deviations, and ranges of the variables) and Pearson’s product-moment correlations to examine the bivariate relationships among the study variables. The PROCESS Macro Version 4.2 [29] is a widely used tool for conducting moderation analyses and offers pre-designed model templates. In alignment with our research objectives, we selected Model 1 from these templates to examine both the direct relationship between CAS and functional impairment (H1) and the moderating effect of gender on this relationship (H2). Both the independent variable (X) and the moderator variable (W), along with their interaction term (X*W), are included as predictor variables in this model. It also allows for the incorporation of control variables into the regression function. This model enables researchers to address multiple questions simultaneously. First, it facilitates the examination of whether there is a significant relationship between the independent and dependent variables while controlling for the effects of the moderator, the interaction term, and the control variables. Second, it allows researchers to evaluate whether the addition of the interaction term leads to a statistically significant increase in the explained variance of the dependent variable. If the interaction term is found to be statistically significant, the strength and significance of the relationship between the independent and dependent variables are re-evaluated across different levels of the moderator variable. This analysis helps to clarify how the moderator affects this association.

In our study, CAS was the independent variable, and gender functioned as the moderator. Impairments in seven distinct domains as well as overall functioning were addressed as dependent variables. Additionally, we incorporated participants’ comorbid symptoms—anxiety, depression, negative self-concept, somatization, and hostility—into the model as control variables. Given that there were eight dependent variables, we conducted eight moderation models for each functional impairment score. In each model, we first examined the direct relationship between CAS and functional impairment as well as the moderating effect of gender on this relationship. If the analyses showed a statistically significant difference in R-square (ΔR-square) for the interaction term, we subsequently analyzed the association between CAS and functional impairment separately for women and men to assess how this relationship differs based on participants’ gender. SPSS Version 23.0 was used for all analyses.

## Results

The analysis sample included 197 men and 483 women. The descriptive statistics for the study variables are presented in Table 1. Participants’ ages ranged from 17 to 30 years, with a mean of 20.87 years (SD = 2.04). The internal reliabilities of the study variables and their bivariate correlations are shown in Table 2. Cronbach’s alphas ranged between .73 and .96, indicating moderate to high reliabilities for most of the scales. Women had higher scores on BSI somatization and WFIRS-S self-concept than men, whereas the opposite was true for WURS-total and WFIRS-S scores for the school, social, and risk domains. As age increased, BSI somatization decreased. However, age was positively correlated with WFIRS-S-total and WFIRS-S scores in the school, work, and life skills domains. WURS-total was positively correlated with comorbid psychiatric symptoms and functional impairment. Positive relationships were also found between all BSI and WFIRS-S scores.

**Table 1:** Descriptive statistics for the study variables.

Variables	Range	Mean	SD
1. Gender^a^	**–**	–	–
2. Age	17–30	20.87	2.04
3. BSI-Total	0–2.87	0.92	0.60
4. BSI-Anxiety	0–3.08	0.80	0.62
5. BSI-Depression	0–3.58	1.23	0.80
6. BSI-Negative self-concept	0–3.75	0.95	0.72
7. BSI-Somatization	0–2.89	0.60	0.53
8. BSI-Hostility	0–3.29	0.93	0.64
9. WURS-Total	0–2.76	0.85	0.57
10. WFIRS-S-Total	0.03–2.56	0.79	0.38
11. WFIRS-S-Family	0–2.63	0.99	0.50
12. WFIRS-S-Work	0–3	0.93	0.61
13. WFIRS-S-School	0–3	0.69	0.51
14. WFIRS-S-Life skills	0–2.58	0.97	0.53
15. WFIRS-S-Self-concept	0–3	1.49	0.79
16. WFIRS-S-Social	0–2.78	0.75	0.48
17. WFIRS-S-Risk	0–2.57	0.23	0.29

*Note*. *N* = 680. The scores were calculated by averaging the item scores on the related scales and sub-scales. SD = Standard Deviation; BSI = Brief Symptom Inventory; WURS = Wender–Utah Rating Scale; WFIRS-S = Weiss Functional Impairment Rating Scale-Self.

^a^Gender: Men = 0, Women = 1.

**Table 2:** Bivariate correlations among the study variables.

	1.	2.	3.	4.	5.	6.	7.	8.	9.	10.	11.	12.	13.	14.	15.	16.	17.
1. Gender^a^	–	−.12^**^	.05	.04	.06	.01	.11^**^	.01	−.12^**^	−.04	.04	.01	−.17^**^	.04	.08^*^	−.12^**^	−.19^**^
2. Age		–	−.02	−.01	−.01	.03	−.09^*^	−.06	.05	.08^*^	.05	.10^**^	.09^*^	.09^*^	.05	.03	.04
3. BSI-Total			(.96)	.94^**^	.94^**^	.92^**^	.78^**^	.81^**^	.61^**^	.74^**^	.47^**^	.56^**^	.55^**^	.66^**^	.71^**^	.59^**^	.37^**^
4. BSI-Anxiety				(.87)	.83^**^	.82^**^	.71^**^	.72^**^	.57^**^	.70^**^	.44^**^	.56^**^	.54^**^	.63^**^	.62^**^	.55^**^	.36^**^
5. BSI-Depression					(.90)	.83^**^	.68^**^	.69^**^	.53^**^	.69^**^	.42^**^	.52^**^	.50^**^	.63^**^	.75^**^	.57^**^	.28^**^
6. BSI-Negative self-concept						(.88)	.60^**^	.69^**^	.57^**^	.69^**^	.42^**^	.52^**^	.52^**^	.59^**^	.70^**^	.57^**^	.33^**^
7. BSI-Somatization							(.77)	.58^**^	.47^**^	.54^**^	.37^**^	.39^**^	.41^**^	.53^**^	.42^**^	.41^**^	.31^**^
8. BSI-Hostility								(.78)	.53^**^	.62^**^	.47^**^	.44^**^	.46^**^	.54^**^	.54^**^	.47^**^	.41^**^
9. WURS-Total									(.90)	.66^**^	.53^**^	.50^**^	.51^**^	.55^**^	.46^**^	.51^**^	.43^**^
10. WFIRS-S-Total										(.96)	.68^**^	.84^**^	.83^**^	.86^**^	.70^**^	.73^**^	.57^**^
11. WFIRS-S-Family											(.73)	.47^**^	.47^**^	.53^**^	.40^**^	.43^**^	.42^**^
12. WFIRS-S-Work												(.88)	.77^**^	.67^**^	.54^**^	.50^**^	.33^**^
13. WFIRS-S-School													(.83)	.64^**^	.46^**^	.51^**^	.47^**^
14. WFIRS-S-Life skills														(.84)	.60^**^	.59^**^	.39^**^
15. WFIRS-S-Self-concept															(.91)	.53^**^	.20^**^
16. WFIRS-S-Social																(.80)	.35^**^
17. WFIRS-S-Risk																	(.84)

*Note*. *N* = 680. Cronbach’s alphas for the scales are shown in parenthesis along the diagonal axis. SD = Standard Deviation; BSI = Brief Symptom Inventory; WURS = Wender–Utah Rating Scale; WFIRS-S = Weiss Functional Impairment Rating Scale-Self.

^a^Gender: Men = 0, Women = 1.

* *p* <.05,

** *p* <.01.

In eight moderation models, we assessed both the relationship between CAS and functional impairment (H1) as well as the moderating effect of gender on this relationship (H2) for both general and domain-specific functional impairment. We added age as another control variable with concomitant psychiatric symptoms (i.e., five BSI scores) because age was significantly correlated with impairment scores. CAS explained the statistically significant variance in functional impairment at both the general and domain-specific levels in all models and demonstrated positive associations with all impairment scores. The interaction term reflecting the relationship between gender and CAS was not statistically significant in explaining impairments across various domains: family (b = –.01, t = –0.12, p =.908), work (b = –.08, t = –1.09, p =.278), school (b = –.10, t = –1.80, p =.073), life skills (b = –.09, t = –1.60, p =.111), and self-concept (b = –.06, t = –0.87, p =.383). This suggests that gender did not moderate the relationship between CAS and dysfunction in these areas. CAS, however, significantly interacted with gender in explaining overall functional impairment (b = –.08, t = –2.26, p =.024). Despite exhibiting a significant effect in both gender groups, the association between CAS and general functional impairment was stronger in men (b =.27, p <.001, CI [.21, .33]) than in women (b =.19, p <.001, CI [.14, .24]). Gender also moderated the link between CAS and impairments in the social (b = –.11, t = –2.00, p =.046) and risk domains (b = –.10, t = –2.86, p =.004). While both genders’ CAS were significantly associated with social domain impairments, this association was stronger for men (b =.25, p <.001, CI [.16, .35]) than for women (b =.15, p <.001, CI [.08, .22]). Similarly, the relationship between CAS and impairments in the risk domain was stronger in men (b =.20, p <.001, CI [.13, .26]) than in women (b =.09, p <.001, CI [.05, .14]). Table 3 presents the regression weights for all variables examined in the eight models, along with model significance and explained variance.

**Table 3:** Examining the moderating role of gender in the relationship between CAS and overall and domain-based functional impairment.

	WFIRS-S-Total		WFIRS-S-Family		WFIRS-S-Work		WFIRS-S-School		WFIRS-S-Life skills		WFIRS-S-Self-concept		WFIRS-S-Social		WFIRS-S-Risk	
**Independent variables**	Coeff.	SE	Coeff.	SE	Coeff.	SE	Coeff.	SE	Coeff.	SE	Coeff.	SE	Coeff.	SE	Coeff.	SE
CAS (X)	.27^***^	.03	.34^***^	.05	.33^***^	.06	.27^***^	.05	.28^***^	.05	.17^**^	.06	.25^***^	.05	.20^***^	.03
Gender (W)	.05	.04	.10	.06	.12	.08	−.08	.06	.13^*^	.06	.19^*^	.08	−.02	.06	−.01	.04
Interaction (X*W)	−.08^*^	.03	−.01	.06	−.08	.07	−.10	.06	−.09	.06	−.06	.07	−.11^*^	.05	−.10^**^	.04
Age	.01^**^	.00	.02	.01	.03^**^	.01	.02^*^	.01	.03^***^	.01	.02	.01	.00	.01	.01	.00
Anxiety	.10^**^	.03	.01	.06	.28^***^	.07	.17^**^	.05	.15^**^	.05	−.07	.07	.05	.05	.02	.03
Depression	.11^***^	.02	.02	.04	.10^*^	.05	.04	.04	.18^***^	.04	.63^***^	.05	.16^***^	.04	−.06^*^	.02
Negative Self-concept	.05	.03	−.01	.05	.04	.05	.06	.04	−.02	.04	.26^***^	.06	.11^**^	.04	.01	.03
Somatization	.00	.03	.02	.04	−.05	.05	.04	.04	.10^*^	.04	−.26^***^	.05	−.02	.04	.06^*^	.03
Hostility	.07^**^	.02	.18^***^	.04	.01	.04	.05	.04	.06	.04	.04	.05	.01	.03	.12^***^	.02
*R*-square	.63		.34		.37		.39		.49		.60		.42		.28	
*F*(9, 670)	126.84^***^		39.06^***^		43.97^***^		47.64^***^		71.97^***^		112.28^***^		53.15^***^		28.72^***^	
ΔR-square - X*W	.003		.000		.001		.003		.002		.000		.004		.009	
ΔF(1, 670) - X*W	5.11^*^		0.01		1.18		3.23		2.55		0.76		4.00^*^		8.20^**^	

*Note*. *N* = 680. WFIRS-S = Weiss Functional Impairment Rating Scale-Self. Gender: Men = 0, Women = 1.

* *p* <.05,

** *p* <.01,

*** *p* <.001.

All models were statistically significant at an alpha level of .001, with explained variance ranging from .28 to .63. However, R-square change values were relatively small, indicating small effect sizes. The inclusion of six control variables, five of which represent comorbid psychiatric symptoms, may have contributed to the variance explained in the models. Notably, despite the small effect sizes, the interaction terms remained statistically significant in several models, indicating that gender moderated the association between CAS and functional impairment in some domains. Table 3 presents the regression coefficients, effect sizes, and explained variance for all models.

CAS demonstrated a positive correlation with functional impairment at both the general and domain-specific levels, supporting our first hypothesis. Gender was identified as a moderating factor influencing the relationship between CAS and general functioning as well as social and risk domains. Specifically, men were found to be more likely than women to experience greater overall functional impairments as well as impairments in social and risk domains linked to their early ADHD symptoms. However, the moderating effect of gender was not confirmed in other areas—family, work, school, life skills, and self-concept. Thus, the findings provided partial support for our second hypothesis.

## Discussion

This study investigated the relationship between CAS and the functional difficulties experienced by university students, with gender considered as a moderating factor. We controlled for the age of the participants and other psychiatric symptoms, including depression, anxiety, somatization, hostility, and negative self-concept. Consistent with our first hypothesis, we found a positive relationship between CAS and functional impairment at both the general and domain-specific levels. Our findings also revealed that CAS were more strongly associated with overall functional impairment in men than in women. Furthermore, men with higher levels of CAS demonstrated a significantly higher level of impairment in social and risk areas compared to women. This confirms the moderating effect of gender on the relationship between CAS and functional impairment in the general, social, and risk areas. However, the strength of the relationship between CAS and functional impairment was unaffected by gender in the family, work, school, life skills, and self-concept domains. These findings offer partial support for our second hypothesis regarding the moderating role of gender.

### CAS and functional impairment

ADHD is a disorder that can cause significant functional impairment [30]. The findings of this study support research showing that ADHD plays a significant role in overall functional impairment [4,5,30]. Longitudinal studies have reported different rates of ADHD diagnosis persistence, ranging from 4% to 76% during the transition to adulthood [31]. Additionally, a study found that a childhood ADHD diagnosis is associated with an 80% rate of functional impairment in adulthood, regardless of whether ADHD symptoms persist at the clinical level [2]. Our study supports these findings and shows that CAS contributes to the general functional impairment observed in university students. Therefore, we suggest that clinical assessments should include an evaluation of both the history of CAS and current psychiatric symptoms to better understand functional impairment, even in university students who have not been diagnosed with ADHD. In addition, the results of our study suggest that well-functioning individuals may still experience impairments due to CAS. Given the moderate positive correlation between CAS and functional impairment scores, we suggest that students, particularly men, with a higher symptom burden are at greater risk for functional impairment and should be evaluated. However, we are unable to comment on the role of treatment, as cases receiving ADHD treatment were not included in the study. Nevertheless, we can conclude that students with higher CAS who were not treated may have higher impairments.

### The moderating effect of gender on overall functional impairment

Several studies have reported that gender can have an effect on ADHD and its outcomes [9,32,33]. While most of these studies have found that men are more strongly affected by ADHD symptoms [9,32], Fedele et al. [33] conducted a large clinical study consisting primarily of females (78%) and found that women are more affected. However, the gender distribution of the sample in that study [33] was inconsistent with previous reports obtained from clinical and community populations [8–10], suggesting that it may not represent the general ADHD profile. Conversely, other studies have emphasized the similarity between men and women in terms of ADHD outcomes [34], although comorbid psychiatric symptoms were not evaluated [33,35] or controlled for [32,34]. Thus, the effect of concomitant psychiatric symptoms on functional impairment could not be eliminated in these studies questioning the association between ADHD and functional impairment.

In our study, a broad range of psychiatric symptoms were screened, allowing for more specific CAS results. Consistent with a large body of evidence [9,32], the results showed that CAS impairs both men and women in university students, but men are more likely to be affected negatively. This highlights the importance of evaluating and treating CAS in clinical practice for children and university students. In addition, clinicians should consider that men are more vulnerable to CAS than women. However, although we found men to be more vulnerable to CAS, there was a significant positive relationship between CAS and functional impairment in our women participants as well. Given studies reporting difficulties in recognizing ADHD in females [8–10], we emphasize the need to assess ADHD symptoms in women during childhood and college years.

One notable aspect of our findings was that, while the moderating effect of gender was statistically significant, the R-square change values remained relatively small. This is consistent with prior research indicating that moderation effects in psychological models often produce small effect sizes [30]. A possible explanation for this is the inclusion of multiple control variables, particularly comorbid psychiatric symptoms, which are known to contribute substantially to functional impairment [17,18]. Given that five of the six control variables in our models represented different psychiatric symptoms, it is likely that their collective influence accounted for a significant portion of the explained variance, thereby reducing the prominence of the interaction term. Despite this, prior literature suggests that even small but significant moderation effects can yield meaningful insights into complex psychological relationships, particularly in applied research settings [30]. Thus, while the effect sizes were modest, they still provide valuable information about the moderating role of gender in CAS-related functional impairment.

### The moderating effect of gender on subdomains of functional impairment

In this study, we observed different results in subdomains of functional impairment in terms of the moderating effect of gender. The associations between CAS and functional impairments in the social and risk domains were stronger in men than in women. Studies have reported varying results regarding the effect of ADHD symptoms on women compared to men in terms of psychosocial functioning. Some have suggested that women are more affected [9], while others have indicated that both genders are similarly affected [34]. In a study that examined psychosocial functioning in adolescents with ADHD, it was found that women were more vulnerable to psychosocial problems than men, particularly internalization symptoms [36]. Conversely, in our sample, the relationship between CAS and social impairment in men was stronger when psychiatric symptoms were controlled, including internalization symptoms. Based on this result, we suggest that men may be more prone to social impairments due to CAS. However, it is important to note that our sample consisted of high-functioning individuals.

Gender was also found to be a moderator in the relationship between CAS and risk-taking behaviors. Previous studies have reported that adults with ADHD are more prone to risk-taking behaviors, such as gambling, substance use, unprotected sex, and careless driving [37–39]. Shoham et al. investigated factors associated with risky behaviors in adults with ADHD and found that externalizing symptoms, which were reported to be more frequent in men [8–10], were significantly and positively associated with risky behaviors that may lead to physical or non-physical negative consequences [40]. This is consistent with our finding that men exhibited higher levels of risky behaviors associated with CAS.

The effects of gender have been investigated for some sub-dimensions of functional impairment (e.g., psychosocial and cognitive) [9,11,12,34,36], and the results of the few studies investigating gender differences in risk-taking behaviors are consistent with our findings [37,38]. Adolescent men with ADHD have been shown to be more likely to engage in risky behaviors [41,42]. Additionally, in an adult ADHD sample, men were found to be more vulnerable to risky behaviors than women. Notably, that study predominantly included participants with low education levels (below secondary school) [43]. In our study, we showed that this tendency among men is also present at higher education levels. Consistent with the literature [37–39], in our sample, both men and women with high CAS had a greater likelihood of engaging in risky behaviors, but the association between CAS and risky behaviors was stronger among men. Therefore, it can be concluded that men are more prone to ADHD symptom-related risky behaviors, even if they have a high educational level.

### Strengths and limitations

The study has several strengths. First, it screened a wide range of current psychiatric symptoms and controlled for them statistically, leading to specific findings related to ADHD symptoms. Second, it involved university students, a high-functioning sample, showing that some impairments persist even in such populations. Third, functional impairment was evaluated using a comprehensive scale. For instance, when assessing the school sub-dimension, various factors such as relationships with authority figures, scheduling difficulties, note-taking, and homework completion were considered, rather than solely focusing on exam grades. Functional impairment was also assessed in multiple subdomains.

However, the study has some limitations as well. First, the data were obtained from self-report scales and were not validated through other sources. Second, the cross-sectional design limits inferences about causality. Third, retrospective assessment of CAS has the potential for bias. Fourth, the sample was predominantly women, likely due to the gender composition of the faculties where the study was conducted. Therefore, the results must be confirmed in more balanced samples in terms of gender distribution. Fifth, gender was assessed through a dichotomous question offering only the options “man” and “woman,” meaning individuals identifying outside of these categories may not have been represented in the study. Finally, as this was a two-center study, the results cannot be generalized to all university students from different universities or departments.

## Conclusions

This study contributes to the existing literature by examining the moderating effect of gender on the relationship between CAS and functional impairment among university students. The findings indicate that CAS is positively associated with functional impairment, even after controlling for current psychiatric symptoms. As hypothesized, gender was found to moderate the relationship between CAS and functional impairment. Both genders may experience impairments related to CAS, although women are typically thought to manage CAS more effectively. However, men appear to be more vulnerable to CAS, particularly in the social, and risky behavior subdomains. Therefore, we recommend that clinicians assess CAS in conjunction with current psychiatric symptoms in university students and consider men’s increased vulnerability to CAS.

## Supporting information

S1 Data The dataset was presented under the caption ‘Data’. (XLSX)
